# Comparative proteomic and transcriptomic analyses provide new insight into the formation of seed size in castor bean

**DOI:** 10.1186/s12870-020-2249-1

**Published:** 2020-01-30

**Authors:** Anmin Yu, Fei Li, Aizhong Liu

**Affiliations:** 1 Key Laboratory for Forest Resources Conservation and Utilization in the Southwest Mountains of China, Ministry of Education, Southwest Forestry University, Kunming, 650224 People’s Republic of China; 2 Key Laboratory of Economic Plants and Biotechnology, Yunnan Key Laboratory for Wild Plant Resources, Kunming Institute of Botany, Chinese Academy of Sciences, Kunming, 650201 People’s Republic of China

## Abstract

**Background:**

Little is known about the molecular basis of seed size formation in endospermic seed of dicotyledons. The seed of castor bean (*Ricinus communis* L.) is considered as a model system in seed biology studies because of its persistent endosperms throughout seed development.

**Results:**

We compared the size of endosperm and endospermic cells between ZB107 and ZB306 and found that the larger seed size of ZB107 resulted from a higher cell count in the endosperm, which occupy a significant amount of the total seed volume. In addition, fresh weight, dry weight, and protein content of seeds were remarkably higher in ZB107 than in ZB306. Comparative proteomic and transcriptomic analyses were performed between large-seed ZB107 and small-seed ZB306, using isobaric tags for relative and absolute quantification (iTRAQ) and RNA-seq technologies, respectively. A total of 1416 protein species were identified, of which 173 were determined as differentially abundant protein species (DAPs). Additionally, there were 9545 differentially expressed genes (DEGs) between ZB306 and ZB107. Functional analyses revealed that these DAPs and DEGs were mainly involved in cell division and the metabolism of carbohydrates and proteins.

**Conclusions:**

These findings suggest that both cell number and storage-component accumulation are critical for the formation of seed size, providing new insight into the potential mechanisms behind seed size formation in endospermic seeds.

## Background

Seeds are vitally important for the economic and nutritional value of most agricultural products. Consequently, improving the traits associated with seed phenotypes has increasingly received attention for its implications in modern agricultural research. Seed size is a major determinant of crop yield and is one of major traits concerned with the breeding of oil crops that is strongly selected for crop domestication [1]. Seed size is largely governed by genetic factors during the seed-filling process, though the formation process of seed size is also strongly affected by biotic and abiotic stresses [2]. Seed-filling is the period when embryogenesis and endosperm genesis occur, a period that encompasses complex cellular processes and the rapid accumulation of seed storage reserves. Although several QTLs or genes (in particular, transcription factors) have been identified and/or cloned from numerous species such as *Arabidopsis* [3], rice [4], and maize [5], the role of a single gene appears to be minor, and little is understood about the regulatory networks that provide global control over the process of seed size formation. Physiologically, the biosynthetic pathways responsible for the accumulation of seed storage reserves are now largely defined [6], but much less remains unknown about the mechanisms that determine different seed sizes during seed-filling.

The seed of castor bean (*Ricinus communis* L., Euphorbiaceae, 2*n* = 20) is an desirable model system for studying seed biology because of the large and persistent endosperm. It is therefore ideal to study the mechanisms behind seed size formation, as the endosperm contains 60% fatty acids and 34% protein [6, 7]. Among all the vegetable oils, castor oil is a highly valued resource in the industry, due to its high ricinoleic acid (over 85%) content, an unusual fatty acid that consists of 18 carbons, a double bond between C9 and C10, and a hydroxyl group attached to C12. Owing to its excellent solubility in either ethanol or methanol, castor oil has been proposed as a source of high-value biodiesel [8–10]. Because of its high economic value and strong capacity for environmental adaptation, castor bean is widely cultivated in tropical, sub-tropical, and warm-temperate countries, particularly in India, China, and Brazil [11]. With the increasing demand for castor oil in many countries, breeding and the genetic improvement of castor bean varieties for both seed and oil yields are attracting much attention from breeders [12]. Seed size is a critical trait associated with crop yield in castor bean and elucidating the molecular mechanisms that underlie the formation of seed size would greatly facilitate the improvement of genetic engineering in castor bean varieties.

Currently, proteomic and transcriptomic sequencing have offered useful approaches to investigate the integrated mechanisms that underlie both the regulatory networks of seed size formation as well as the accumulation of carbohydrates, proteins, and fatty acids [13]. Recently, iTRAQ technology has also offered a useful quantitative proteomic method to study the seed development in many plant species such as wheat, soybean, rapeseed, and *Medicago truncatula* [14–17]. Differential proteomic analysis on developing castor bean seeds has been conducted with two goals. The first is to identify proteins involved in the biosynthesis of fatty acids in the endosperm of castor bean [6]. The second is to investigate the spatial and temporal trends of protein abundances associated with protein synthesis and degradation in the maternal seed tissues of nucellus [18]. So far, the analysis of embryogenesis, endosperm genesis, and the primary metabolisms during the seed development of castor bean has deserved little attention. In this study, we performed comparative proteomic and transcriptomic analyses on developing seeds from the two inbred varieties ZB107 and ZB306 that have different seed sizes using iTRAQ and RNA-seq techniques. The aim of this study was to identify the candidate genes involved in the formation of seed size at both the levels of transcription (mRNA) and translation (protein), as well as provide novel insights to understand the potential molecular basis that regulates the formation of seed size in castor bean.

## Results

### Morphological and weight changes in seeds during development

**Fig. 1:**
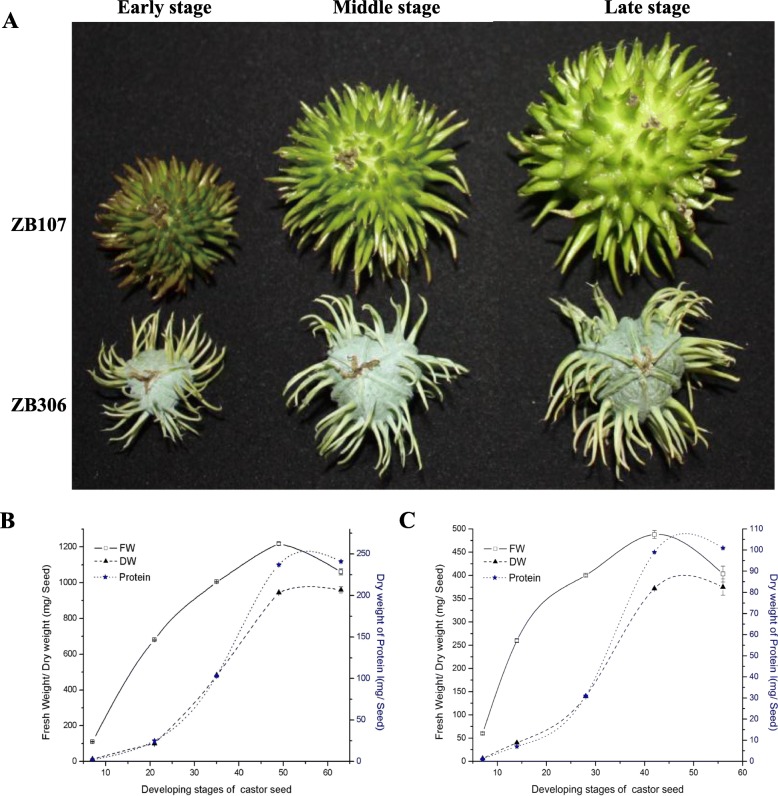
Characterization of castor bean seed development for large-seed ZB107 and small-seed ZB306. **a** Morphology of ZB107 and ZB306 fruits in the early, middle, and late stages. **b, c** The fresh weight, dry weight, and protein content (Protein) of large-seed ZB107 and small-seed ZB306 during seed development. The *white square* represents fresh weight (FW), the *black triangle* represents dry weight (DW), and the *blue asterisk* represents the protein content (Protein). The data were derived from three biological replicates. Each *point* represents the mean ± SD

The length of time of seed development, beginning with pollination to maturation, may exert significant influence on castor varieties, in accordance with the morphological changes of endosperm and our previous study of seed coat development [19, 20]. The seed development process could be separated into three stages: early, middle, and late. Seeds progressed through these three stages in 1–25, 26–45, and 46–75 days after fertilization (DAF) in ZB107, as well as in 1–15, 16–30, and 31–60 DAF in ZB306, respectively (Fig. 1a). The early stage was characterized by active cell division and was associated with increased cell number in the young seeds. In the middle stage, cell enlargement caused the seed weight and volume to increase rapidly (Fig. 1a). During the late stage, fresh weight (FW) of single seed peaked and then gradually decreased. From early to late stages, FW of single seed increased 8.5-fold in ZB107 (from 142.87 ± 2.27 mg to 1207.03 ± 0.2 mg), whereas FW increased 6.93-fold in ZB306 (from 70.53 ± 0.87 mg to 484.87 ± 0.71 mg) (Fig. 1b and c). Correspondingly, dry weight (DW) of single seed increased 38.5-fold from the early to late stages in ZB107 (from 24.9 ± 0.56 mg to 924.87 ± 0.47 mg), and a 35.45-fold increase (from 10.97 ± 0.35 mg to 390.23 ± 0.97 mg) was observed in ZB306 (Fig. 1b and c). The protein content of dry single seed exhibited a similar trend during seed development, increasing from 6.2 ± 0.36 mg to 285.1 ± 0.4 mg in ZB107, and from 3.1 ± 0.26 mg to 97.5 ± 0.75 mg in ZB306. Similarly, seed size also increased rapidly from the early to late stages in castor bean (Fig. 1a). Seed length increased from 18.85 mm to 37.7 mm in ZB107, and it increased from 16.9 mm to 26 mm in ZB306. Seed width increased from 13 mm to 26 mm in ZB107, and it increased from 11.7 mm to 16.5 mm in ZB306.

**Fig. 2:**
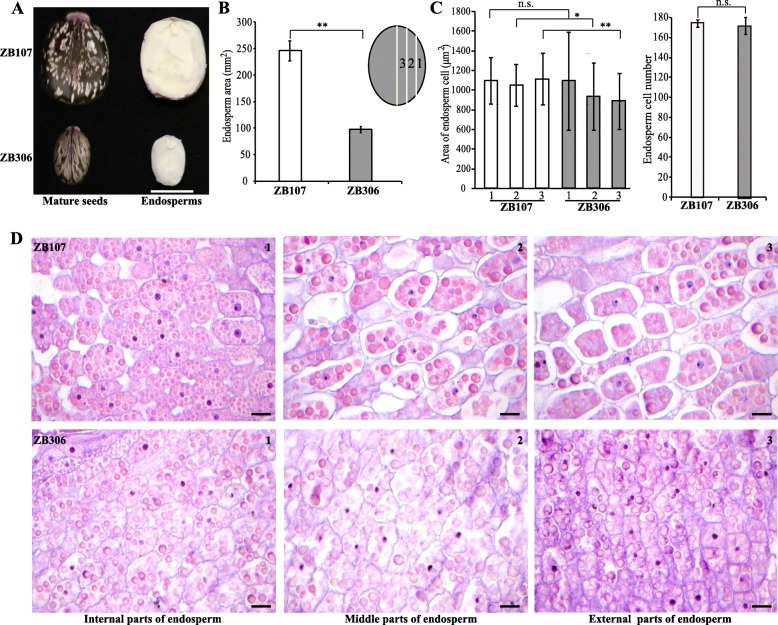
The morphological and cytological characteristics of large-seed ZB107 and small-seed ZB306. **a** The exact appearances of mature seeds and endosperms of ZB107 and ZB306. Scale bar = 1 cm. **b** Comparison of the surface area of endosperm between ZB107 and ZB306. **c, d** Histological analysis of the endosperm cell area and cell number of ZB107 and ZB306. **e** Cross-sections of the endosperm was divided into internal (1), middle (2), and external (3) parts. Data are reported as mean ± SD from 10 seeds. The significance was analyzed using Student *t*-test (The asterisk indicates significance, * *p* < 0.05, ** *p* < 0.01, n.s., no significant difference). Scale bar = 25 μm

As a typical dicot endospermic seed, the endosperm occupies roughly 90% of the volume of the castor bean seed, so the differences in seed size and weight of ZB107 and ZB306 depend mainly on endosperm size. The endosperm area of ZB107 was nearly 2.51-fold larger than ZB306 (Fig. 2a and b). To further investigate whether endosperm size variation was determined by cell size or cell number, we performed a microscopic analysis of the endosperm tissues of ZB107 and ZB306. The variation of cell size and cell number were statistically insignificant between ZB107 and ZB306; however, cell density varied among the internal, middle, and external parts of the two varieties (Fig. 2a, c, d and e). These results indicate that the difference in endosperm size between large-seed ZB107 and small-seed ZB306 is determined by cell number rather than cell size, which is consistent with our previous observations of seed coat in castor bean, in which we noted that large seed coat area resulted from more cell numbers [19].

### Comparative proteomic analysis of large-seed ZB107 and small-seed ZB306

To investigate the protein species involved in seed size formation in castor bean, we performed iTRAQ analysis on large-seed ZB107 and small-seed ZB306. The seed samples of ZB107 from the early, middle, and late stages were used to extract protein, and equal amounts of protein samples from different development stages were mixed as the large-seed sample, and the corresponding seeds of ZB306 in the above development stages were mixed as the small-seed sample. A total of 156,386 spectra were generated, of which 12,674 were matched to the Peptide Spectrum Match (PSM), and 10,506 were unique after eliminating low-score spectra (Additional file 1: Table S1). By searching against the reference genome database with the Mascot 2.2 program, these unique spectra that met the strict confidence criteria were matched with 3963 known peptides, of which 3681 peptides were unique (Additional file 1: Table S1). Finally, 1416 protein species containing at least one unique peptide were identified from the developing seeds of ZB107 and ZB306 (Additional file 2: Table S2). Of the 1416 protein species, 1028 were divided into 23 COG clusters according to their functional annotation (Additional file 3: Figure S1). Among these, “posttranslational modification/protein turnover/chaperones” was the functional category with the highest representation (17.41%, 179/1028), followed by “general function prediction only” (14.40%, 148/1028), “translation/ribosomal structure/biogenesis” (11.67%, 120/1028), and “energy production and conversion” (10.41%, 107/1028). Furthermore, eight protein species were classified into “cell cycle control, cell division, or chromosome partitioning”, and six of them were identified as calmodulin or calcium-dependent protein kinase (Additional file 4: Table S3). In addition, 1157 identified protein species were categorized into three groups based on GO category enrichment analysis: biological process, cellular component, and molecular function. Results indicated that in the biological process category, metabolic process (60.41%, 699/1157) was the most represented, followed by cellular process (55.92%, 647/1157) and response to stimulus (35.70%, 413/1157) (Additional file 5: Figure S2). The largest category is related to cell and cell part (82.80%, 958/1157) in cellular component. Specifically, three protein species related to cell division were identified, including auxin-repressed 12.5 kDa protein (ARP, 30190.t000561), serine carboxypeptidase (SC, 29489.t000001, 29,745.t000011), and eukaryotic translation initiation factor 5a (eIF-5A, 29,687.t000001) (Table 1).

**Table 1:** Identification of DAPs between ZB107 and ZB306

Metabolic pathway	Protein ID	Description	FC
TCA cycle	29,600.t000017	aconitase, putative	−0.6
	29,794.t000105	malic enzyme	−0.62
	30,226.t000050	citrate synthase (CS)	−0.48
	29,628.t000002	isocitrate dehydrogenase (IDH)	0.72
Glycolysis	27,383.t000001	triosephosphate isomerase (TPI)	−0.64
	29,601.t000014	pyruvate decarboxylase	−0.41
	29,660.t000032	fructose-bisphosphate aldolase (FBA)	−0.64
	29,692.t000012	phosphoglucomutase (PGM)	−0.65
	30,169.t000047	glyceraldehyde 3-phosphate dehydrogenase (GAPDH)	−0.6
	30,169.t000073	phosphoglycerate kinase (PGK)	−0.6
	30,131.t000450	pyruvate kinase (PK)	−0.56
	30,147.t000451	enolase	−0.65
	29,736.t000093	pyruvate dehydrogenase (PDC)	−0.6
	30,170.t000437	glucose-6-phosphate isomerase (GPI)	−0.59
	29,739.t000129	sucrose synthase (SUS)	−0.69
Fructose and mannose metabolism	27,766.t000004	Xylose isomerase	−0.67
	29,842.t000024	phosphofructo kinase (PFK)	−0.63
	30,171.t000020	utp-glucose-1-phosphate uridylyltransferase	−0.83
	27,914.t000023	NADP-specific isocitrate dehydrogenase (IDH)	−0.62
Biosynthesis of amino acids	30,078.t000071	s-adenosylmethionine synthetase	−0.20
	30,147.t000451	enolase	−0.65
	29,739.t000101	26S protease regulatory subunit	−0.58
	29,172.t000012	xylem serine proteinase 1 precursor	−0.52
	29,986.t000074	xylem serine proteinase 1 precursor	−0.57
Cell division	29,489.t000001	serine carboxypeptidase, putative	4.09
	29,745.t000011	serine carboxypeptidase, putative	−0.50
	29,687.t000001	eukaryotic translation initiation factor 5a	−0.54
	30,190.t000561	Auxin-repressed 12.5 kDa protein	2.24
Ribosome	28,180.t000008	60S ribosomal protein L23a (SRPL23a)	−0.29
	29,070.t000002	60S ribosomal protein L32 (SRPL32)	−0.48
	29,703.t000077	60S ribosomal protein L6 (SRPL6)	−0.41
	29,743.t000009	60S ribosomal protein L8 (SRPL8)	−0.54
	30,071.t000004	60S ribosomal protein L13 (SRPL13)	−0.61
	30,128.t000077	40S ribosomal protein S3a	−0.58
	30,147.t000153	60S ribosomal protein L27a (SRPL27a)	−0.45
	30,147.t000458	40S ribosomal protein S13 (RPS13)	−0.56
	30,147.t000462	40S ribosomal protein S20 (RPS20)	−0.62
	30,152.t000032	60S ribosomal protein L6 (SRPL6)	−0.55
	28,076.t000001	60S ribosomal protein L12 (SRPL12)	1.75
	29,588.t000012	40S ribosomal protein S12	2.73
	29,630.t000028	40S ribosomal protein S19	2.39
	29,788.t000001	60S ribosomal protein L35 (SRPL35)	2.04
	29,805.t000016	40S ribosomal protein S23	7.52
	29,866.t000003	60S acidic ribosomal protein P1	7.93
	30,167.t000025	40S ribosomal protein S19	2.48

FC, the log2 fold changes of ZB306/ZB107

**Fig. 3:**
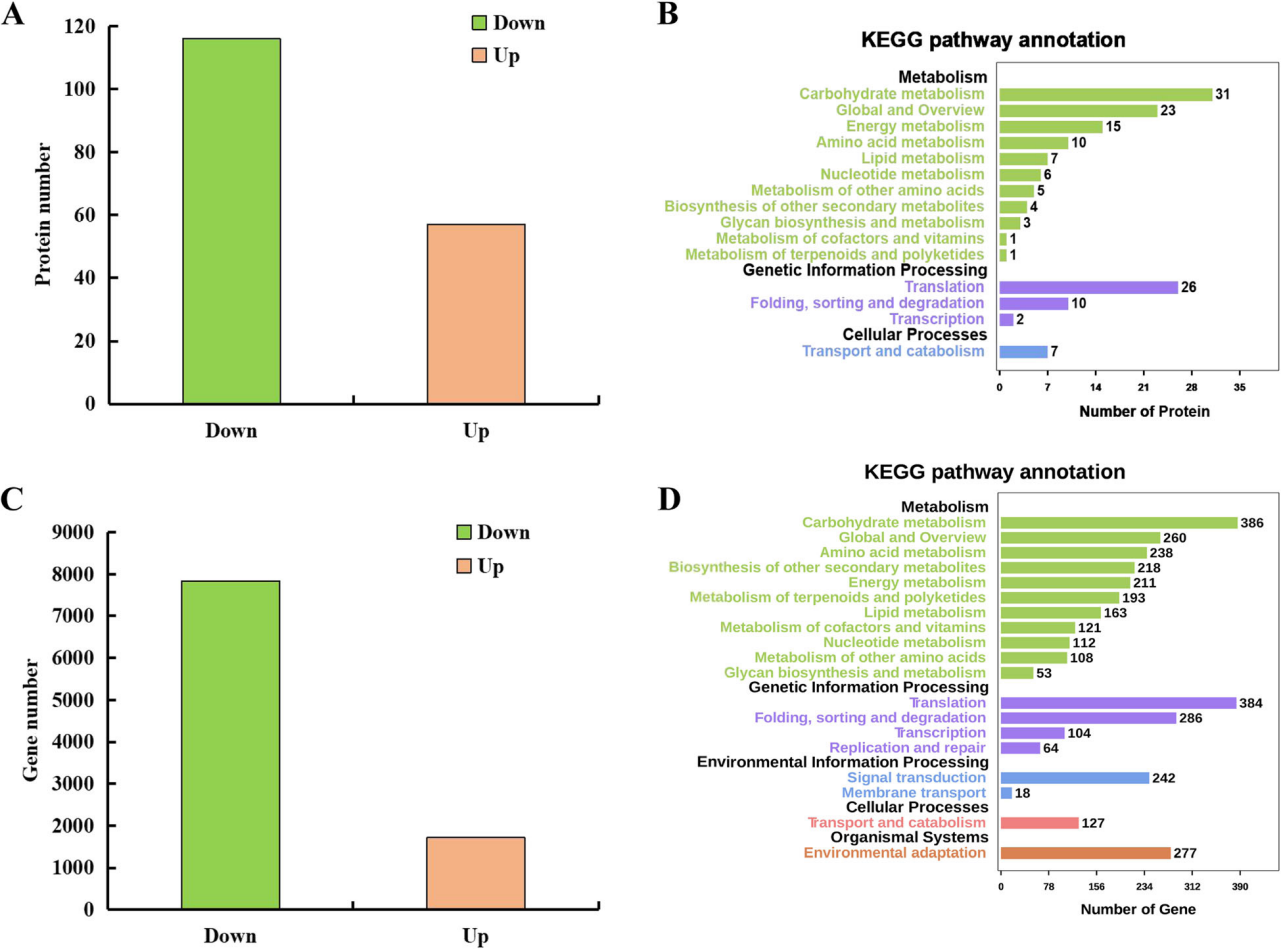
Comparative proteomic and transcriptomic analyses of DAPs and DEGs between large-seed ZB107 and small-seed ZB306. **a, c** The number of increased-abundance (Up) and decreased-abundance (Down) protein species and genes in ZB306 compared to ZB107. **b, d** KEGG pathway annotation of DAPs and DEGs

**Fig. 4:**
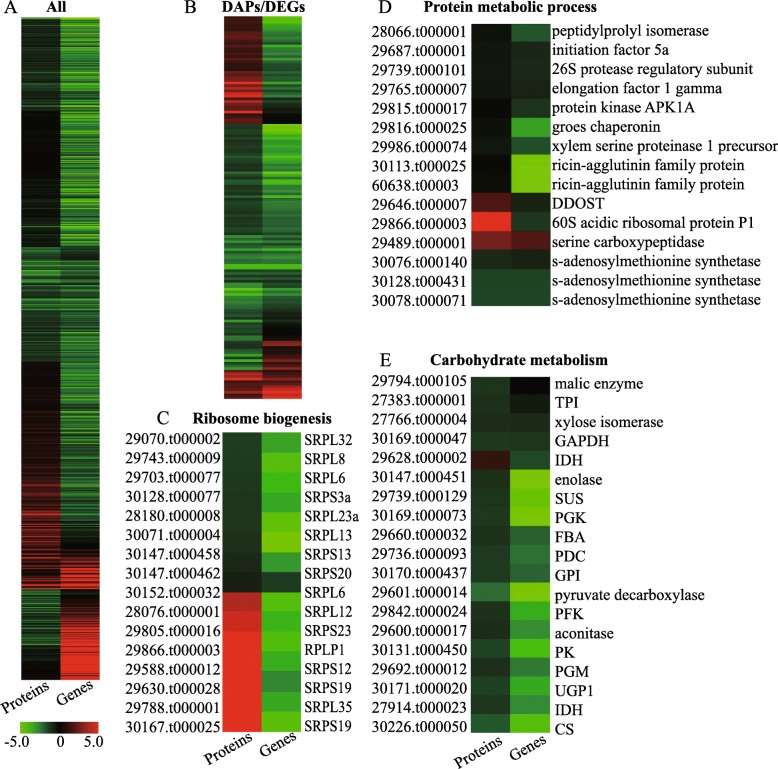
Hierarchical clustering analysis of the proteomic and transcriptomic data. **a** Heatmap of all 1416 quantified protein species and their corresponding mRNAs. **b** Heatmap of all 173 DAPs and their corresponding mRNAs. **c, d, e** Heatmap of the protein species and corresponding mRNAs related to ribosome biogenesis, protein metabolic process, and carbohydrate metabolism. DDOST, dolichyl-diphosphooligosaccharide-protein glycosyltransferase. Log_2_ fold change of protein species abundance (left) and gene expression (right) between ZB107 and ZB306 is presented with different colors: *red* represents up-regulated and *green* represents down-regulated

Using a fold change ≥1.5 or ≤ 0.67 and a *p*-value less than 0.05, a total of 173 protein species were identified as differentially abundant protein species (DAPs) between ZB107 and ZB306. Of these DAPs, 57 increased-abundance and 116 decreased-abundance protein species were detected in small-seed ZB306 compared to large-seed ZB107 (Fig. 3a and Additional file 6: Table S4). Furthermore, KEGG pathway enrichment analysis revealed that a total of 105 DAPs were featured high enrichment in “carbohydrate metabolism” (29.52%, 31/105), “energy metabolism” (14.29%, 15/105), “amino acid metabolism” (9.52%, 10/105) and translation (24.76%, 26/105), respectively (Fig. 3b). These results indicated that protein and carbohydrate metabolism were essential for the determination of seed size/weight of the two castor varieties. In the present study, a total of 19 DAPs involved in carbohydrate metabolism were identified, such as the tricarboxylic acid (TCA) cycle, glycolysis, as well as fructose and mannose metabolism (Table 1). Carbohydrate metabolism is a principal process for the rapid expansion of seed volume, especially in the early developmental stage, and the abundance of nearly all the DAPs involved in this process featured decreased levels in ZB306 (Table 1 and Fig. 3). In the TCA cycle, the abundance of aconitase (29,600.t000017), malic enzyme (29,794.t000105), and citrate synthase (CS, 30226.t000050) featured decreased levels in ZB306 compared to ZB107 (Additional file 7: Figure S3). Likewise, protein abundances of the enzymes involved in glycolysis were also decreased in ZB306 when compared to ZB107, such as triosephosphate isomerase (TPI, 27383.t000001), fructose-bisphosphate aldolase (FBA, 29660.t000032), phosphoglucomutase (PGM, 29692.t000012), and glyceraldehyde 3-phosphate dehydrogenase (GAPDH, 30169.t000047) (Table 1 and Additional file 7: Figure S3). Moreover, seven large ribosomal subunit proteins (28,180.t000008, 29,070.t000002, 29,703.t000077, 29,743.t000009, 30,071.t000004, 30,147.t000153, and 30,152.t000032) that are associated with the ribosomal process exhibited decreases in ZB306, while only three of 40S ribosomal proteins (30,128.t000077, 30,147.t000458, and 30,147.t000462) were decreased-abundance protein species in ZB306 (Table 1 and Fig. 4c). Ribosome biogenesis play a fundamental role in cell growth by activating protein synthesis [21]. In addition, the protein species abundances of the 26S protease regulatory subunit (29,739.t000101) and xylem serine proteinase (29,172.t000012 and 29,986.t000074) associated with the protein metabolic process were also decreased in ZB306 (Table 1 and Fig. 4d). Taken together, the protein species abundances of these enzymes involved in carbohydrate and protein metabolism were lower in small-seed ZB306 than in large-seed ZB107. These results suggested that greater energy and storage reserves are needed for larger seed during the process of embryogenesis and endosperm genesis, resulting in a higher seed weight and larger seed size of ZB107.

**Fig. 5:**
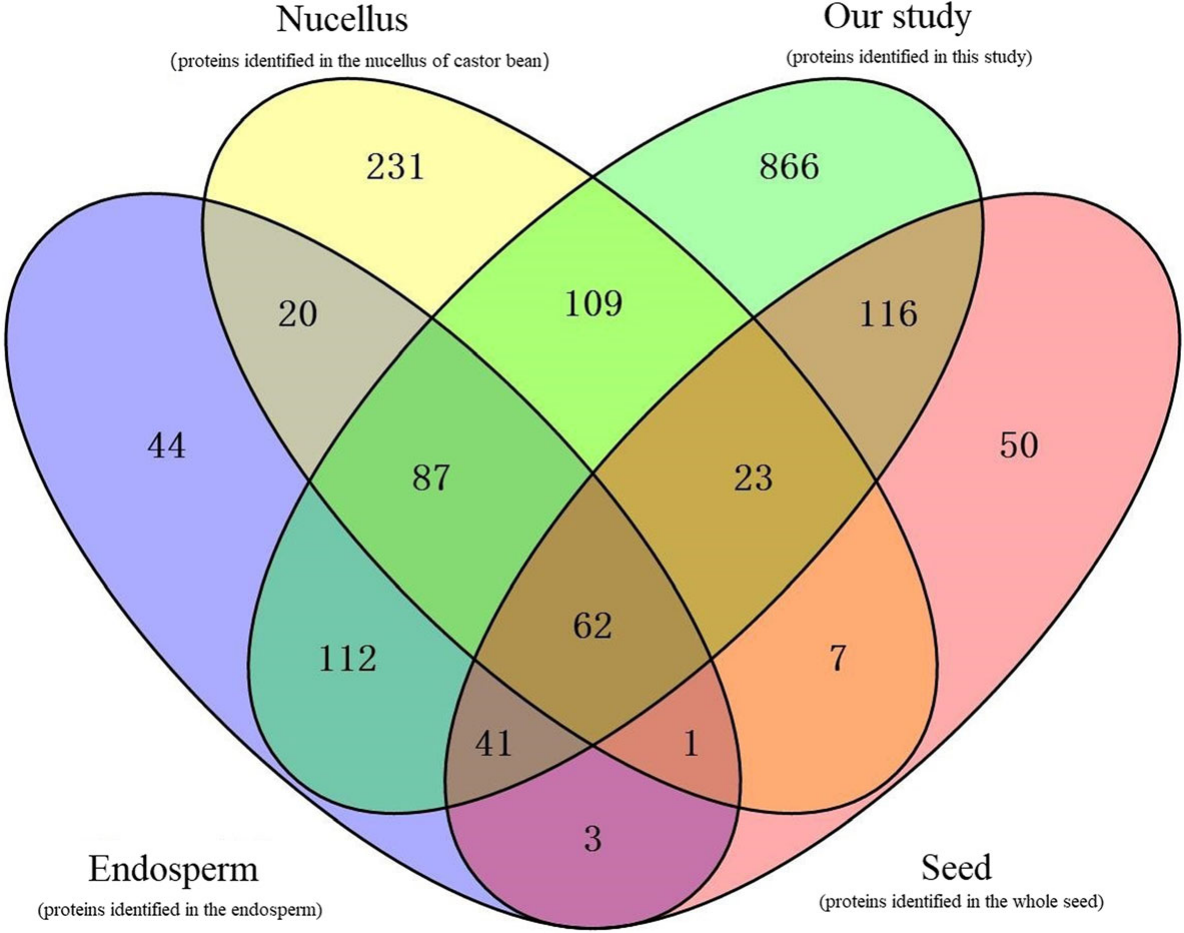
Venn diagram showing the number of protein species identified in previous and current studies. Our study represents the protein species identified in this study; Seed represents protein species identified by Houston et al. [6]; Nucellus and Endosperm represent protein species identified by Nogueira et al. [18, 22]

Furthermore, we compared the protein species identified in this study with the set of protein species previously reported by Houston et al. [6] (a study that generated 522 protein species in developing seeds using two-dimensional gel electrophoresis) and Nogueira et al. (a study that identified 766 and 416 protein species from nucellus and endosperm at different developmental stages, respectively) [18, 22]. A total of 242 protein species were presented in both Houston’s study (using whole seeds, denoted as “Seed”) and our study as shown in Fig. 5. There was an overlap of 302 protein species between our data and Nogueira’s study (only using the endosperm of castor seed, denoted as “Endosperm”), and there was an overlap of 281 protein species between our study and the Nogueira’s research of nucellus (denoted as “Nucellus”). Of note is that a total of 866 new protein species were detected in our study. These protein species were associated with embryogenesis (late embryogenesis abundant protein, 30,128.t000107/29889.t000167), seed coat development (cellulose synthase A catalytic subunit 6, 29,848.t000205), seed storage proteins synthesis (2S albumin precursor, 28,166.t000037/28166.t000041/28166.t000042), and fatty acid synthesis (Long-chain-fatty-acid CoA ligase, 29,732.t000015/29908.t000237) (Additional file 8: Table S5). Furthermore, protein species involved in plant hormone signal transduction pathways also played prominent roles in the embryogenesis/endosperm genesis processes, such as indole-3-acetic acid (IAA)-amido synthetase GH3.5 (28,355.t000001), ARP (30,190.t000561), and so on. These results demonstrate that proteomic analysis is not only capable of both providing a holistic understanding of seed development in castor bean and identifying the largest number of protein species that play crucial roles in the regulation of embryogenesis and endosperm genesis, but also capable of leading us to further important insights into the accumulation of storage products. Most previous studies only used part of the seed, while our study included the whole seeds of ZB107 and ZB306 at three different developmental stages. Taken together, our comparative proteomic analyses revealed that the protein species that participated in seed size variation were enriched by carbohydrate metabolism and protein synthesis during seed embryogenesis and endosperm genesis. This provided a more comprehensive understanding of the molecular basis that underlies seed size control in castor bean.

### Comparative transcriptomic analysis of large-seed ZB107 and small-seed ZB306

**Fig. 6:**
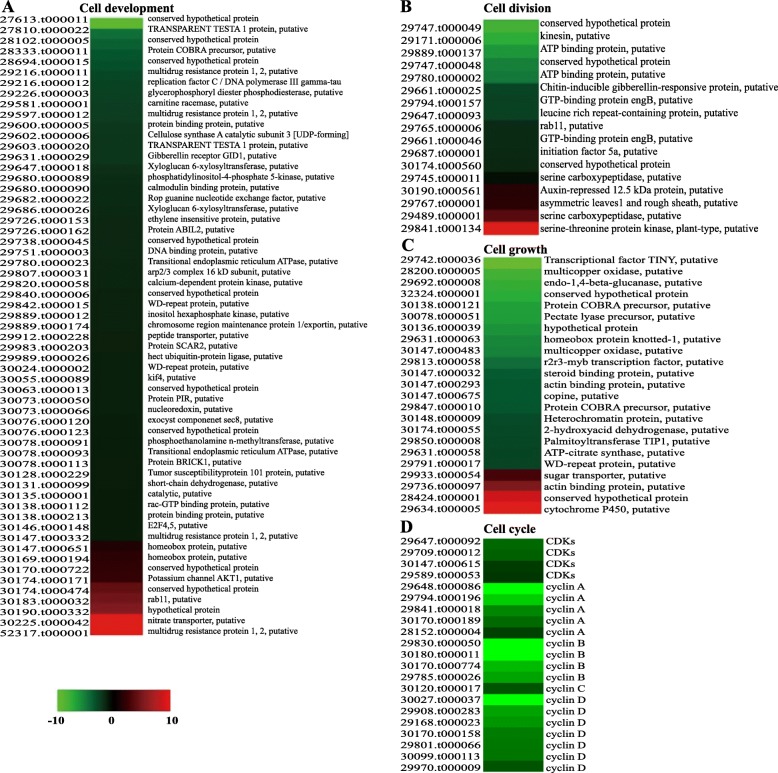
Heatmaps of the DEGs involved in the processes of cell development (**a**), cell division (**b**), cell growth (**c**), and cell cycle (**d**). For each gene, relative expression (ZB306 versus ZB107) is represented in log_2_ fold change. *Red* represents up-regulated and *green* represents down-regulated. CDKs, cyclin-dependent kinases

To investigate the differences in global gene expression during the formation of seed size, RNA-seq analysis was performed using large-seed ZB107 and small-seed ZB306. A total of 49.18 million and 50.65 million high-quality reads from ZB107 and ZB306 were generated, and 79.16 and 84.78% clean reads were mapped to the reference genome of castor bean, respectively. All the uniquely mapped reads were transformed into RPKM to determine the expression level of each transcript, and a total of 9545 DEGs between ZB107 and ZB306 were identified, including 1713 up-regulated and 7832 down-regulated genes in ZB306 compared to ZB107 (Fig. 3c). 386, 238, 163, 384, and 242 DEGs were enriched in the KEGG pathways of carbohydrate metabolism, amino acid metabolism, lipid metabolism, translation, and signal transduction, respectively (Fig. 3d). Carbohydrate and amino acid metabolism were the foremost processes during seed development [14]. Most DEGs involved in the two processes were down-regulated in small-seed ZB306 compared to large-seed ZB107 (Fig. 4 and Additional file 7: Figure S3). For example, the four genes that encode sucrose syntheses (SUS, 29726.t000198, 29,739.t000129, 29,951.t000003, and 29,660.t000014) and are involved in the carbohydrate metabolism process were identified, and three of them showed up-regulation in large-seed ZB107. The higher expression levels of SUS suggested that the content of hexoses is higher in large-seed ZB107 than in small-seed ZB306. It is likely that sugar metabolism in the developing castor bean seeds has many vital functions, such as supplying carbon to the developing endosperm, controlling cell division, or regulating cell differentiation. In addition, the DEGs involved in translation and protein metabolic process were also up-regulated in large-seed ZB107 (see in Fig. 4c and d), such as SRPL32 (29,070.t000002), SRPL8 (29,743.t000009), eIF-5A (29,687.t000001), and S-adenosylmethionine synthetase (SAMS, 30076.t000140, 30,078.t000071, 30,128.t000431). The eIF-5A protein is originally identified as a translation initiation factor, functionally involved in the regulation of cell proliferation, cell growth and cell death [23, 24]. The SAMS is functionally involved in the regulation of methionine metabolism and carbon metabolism [25, 26]. Furthermore, GO enrichment analysis of DEGs showed that 59, 17, 23, and 21 DEGs were enriched under GO terms of cell development (GO:0048468), cell division (GO:0051301), cell growth (GO:0016049), and cell cycle process (GO:0022402), which were associated with cell size and cell number (Fig. 6). Under the cell division term, the gene expression level of kinesin (29,171.t000006) was 5.85-fold lower in ZB306 than in ZB107, while chitin-inducible gibberellin-responsive protein (CIGR, 29661.t000025) and eIF-5A (29,687.t000001) showed 2.19-fold and 1.30-fold down-regulation in ZB306 (Fig. 6b). The CIGR (a member of the GRAS family) is functionally involved in the regulation of cell division, elongation and expansion during seed development [27]. Furthermore, the transcript abundance of TRANSPARENT TESTA 1 protein (TT1, 30,169.t000194) as well as the protein COBRA precursor (29,889.t000012) was 4.49-fold and 3.45-fold lower in ZB306 than ZB107 in the process of cell development (Fig. 6a and c). The COBRA, encoding a putative glycosylphosphatidylinositol-anchored protein, is functionally involved in regulating cellulose synthesis, participated in the formation of plant cell wall [28]. Additionally, the conserved regulators of cell cycle, such as cyclin A (29,648.t000086, 29,794.t000196, 29,841.t000018, 30,170.t000189, 28,152.t000004), cyclin B (29,830.t000050, 30,180.t000011, 30,170.t000774, 29,785.t000026), and cyclin D (30,027.t000037, 29,908.t000283, 29,168.t000023, 30,170.t000158, 29,801.t000066, 30,099.t000113, 29,970.t000009), were also down-regulated in ZB306 (Fig. 6d). Clearly, most of the genes involved in cell division were down-regulated in small-seed ZB306, which strongly suggested that cell number is a critical factor in the regulation of seed size/weight in castor bean.

**Fig. 7:**
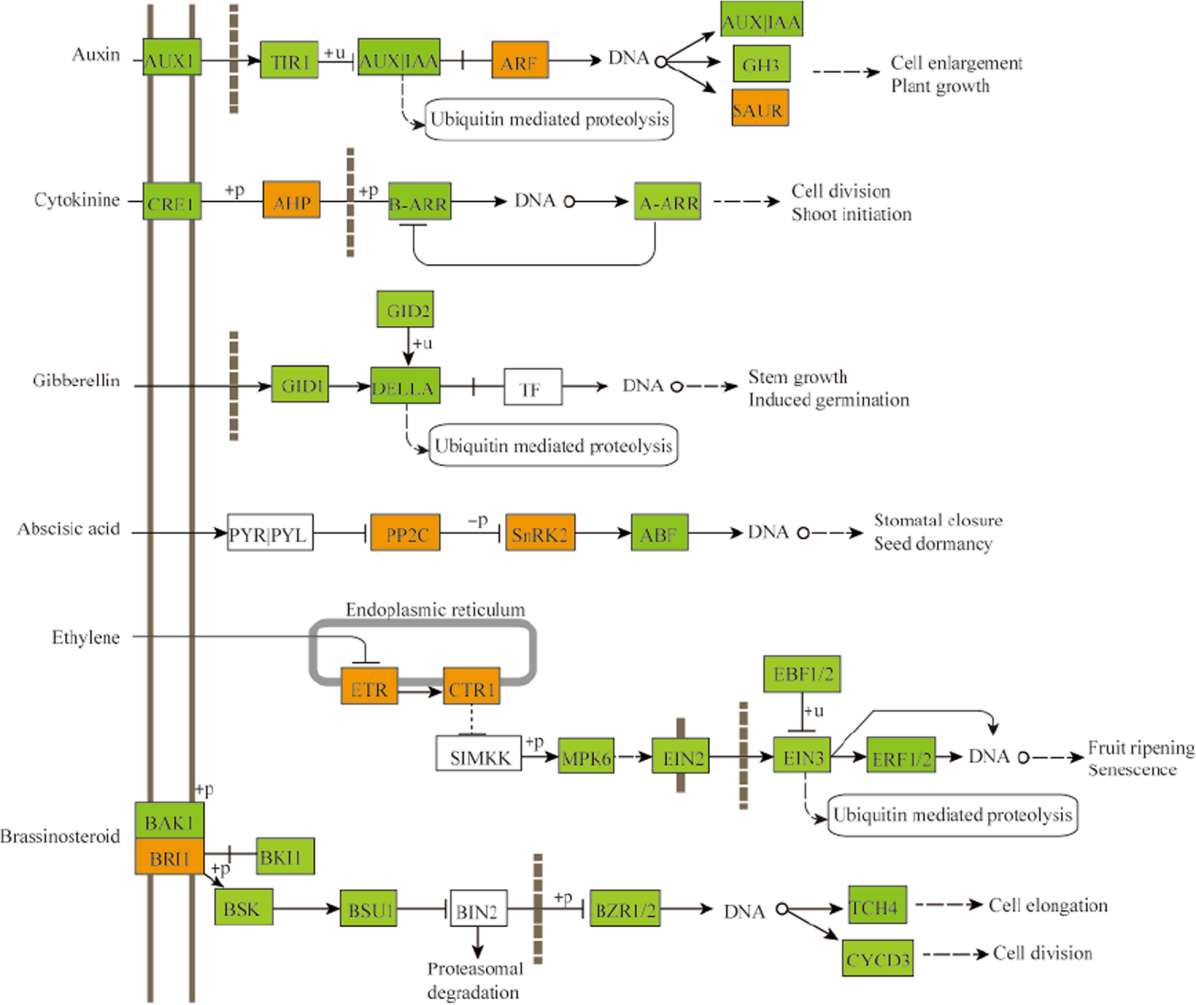
DEGs involved in the plant hormone signal transduction pathways. This graph was modified from a KEGG map (ko04075) according to reference [29]. *Yellow* boxes represent significant up-regulated genes; *green* boxes represent significant down-regulated genes

Plant hormones usually play critical roles in controlling plant growth and development in diverse processes [29]. In the auxin-signaling pathway, the gene that encodes auxin receptors transport inhibitor response 1 protein (TIR1, 29,908.t000274) showed a 2.42-fold higher expression in ZB107. Similarly, genes that encode auxin/indole-3-acetic acid (AUX|IAA, 28179.t000006) and GH3 (28,152.t000001) increased 2.03 and 1.80-fold in ZB107 compared with ZB306 (in Table 2 and Fig. 7). However, the expression level of ARF2 (auxin response factor 2, 29,647.t000040) was found to be significantly higher in ZB306. Consistently, the gene expression of AHP4 (Arabidopsis histidine-containing phosphotransfer protein 4, 29,912.t000167) in cytokinins signaling pathway was up-regulated in small-seed ZB306 (Fig. 7). However, the gene expression of BRI1 interacting proteins in the signaling pathways of brassinosteroids (BRs), such as BRI1-associated receptor kinase 1 (BAK1, 30,190.t000257), BRI1 kinase inhibitor 1 (BKI1, 30,170.t000310), and BR-signaling kinase (BSK, 30190.t000453), all exhibited decreased transcript abundances in ZB306 compared to ZB107 (in Fig. 7). Overall, these observations suggest that plant hormones, i.e., auxin, cytokinin and BR, exert strong influences on the seed development process, which may be an important reason for the difference in seed size between ZB107 and ZB306. To explore the rationale of plant hormones on seed development, we performed a comprehensive *cis*-element analysis for the promoters of all 41 genes involved in hormone signal transduction pathways. We observed that numerous *cis*-elements involved in response to auxin, ABA, GA, MeJA, and SA signals were identified in the promoter regions of these genes. In particular, ten of these genes contained *cis*-elements functionally involved in regulating seed development (such as endosperm-specific expression, seed-specific regulation and cell cycle regulation) (Additional file 9: Figure S4). These *cis*-elements might be related to regulate gene expression in controlling the formation of seed size. Overall, comparative transcriptomic results indicated that cell division was responsible for the higher cell number and larger seed volume in ZB107, while the processes of carbon and protein substance metabolism, as well as hormone signal transduction during embryogenesis/endosperm genesis, were associated with the higher seed weight of ZB107.

**Table 2:** Gene expression of plant hormones related genes

Pathway	Gene name	Gene ID	ZB107 (RPKM)	ZB306 (RPKM)	log2 FC	P-value	FDR
Auxin	AUX1	29,741.t000002	0.86	3.68	2.1	9.58E-20	2.23E-19
	AUX1	28,883.t000036	80.12	0.86	−6.54	0	0
	TIR1	29,908.t000274	30.59	5.71	−2.42	9.34E-230	1.07E-228
	AUX|IAA	29,844.t000020	1.26	3.45	1.46	3.59E-05	5.60E-05
	AUX|IAA	28,179.t000006	232.36	56.82	−2.03	0	0
	ARF10	27,538.t000014	74.25	12.3	−2.59	0	0
	ARF2	29,647.t000040	21.7	74.29	1.78	0	0
	GH3	28,152.t000001	394.07	113.06	−1.8	0	0
	SAUR	29,838.t000091	0.18	43.59	7.92	2.44E-139	1.84E-138
	SAUR	29,739.t000134	40.68	12.65	−1.69	5.39E-50	1.95E-49
CTK	CRE1	29,005.t000011	101.67	2.08	−5.61	0	0
	AHP4	29,912.t000167	18.47	120.46	2.71	6.50E-248	8.01E-247
	AHP1	29,726.t000066	91.15	26.59	−1.78	3.85E-113	2.44E-112
	B-ARR	29,950.t000033	21.97	0.44	−5.63	4.71E-215	5.11E-214
	A-ARR	29,908.t000174	47.41	20.25	−1.23	2.01E-51	7.43E-51
GA	GID1	29,686.t000008	28.97	3.86	−2.91	2.58E-142	1.97E-141
	GID2	30,170.t000622	40.12	12.36	−1.70	3.00E-55	1.16E-54
	DELLA	29,807.t000012	71.54	8.52	−3.07	0	0
ABA	PP2C	30,075.t000035	0.07	273.45	12.01	0	0
	PP2C	30,128.t000337	69.77	14.66	−2.25	0	0
	SnRK2	28,725.t000009	175.59	66.55	−1.40	0	0
	SnRK2	29,908.t000118	10.86	22.02	1.02	3.62E-27	9.62E-27
	ABF	29,801.t000121	55.02	15.19	−1.86	1.35E-201	1.39E-200
Ethylene	ETR	28,802.t000007	49.56	19.23	−1.37	1.09E-198	1.10E-197
	ETR	29,680.t000086	21.25	87.53	2.04	2.69E-155	2.20E-154
	CTR1	29,678.t000014	43.32	10.27	−2.08	8.75E-205	9.11E-204
	CTR1	30,147.t000229	18.26	38.74	1.09	1.08E-53	4.08E-53
	MPK6	29,688.t000016	47.68	20.31	−1.23	3.95E-63	1.65E-62
	EIN2	30,078.t000113	76.78	25.36	−1.60	0	0
	EIN3	30,169.t000172	20.42	9.36	−1.13	5.01E-53	1.88E-52
	EBF1/2	29,848.t000185	130.53	16.02	−3.03	0	0
	ERF1/2	30,147.t000602	1.74	0.59	−1.56	0	0
BR	BAK1	30,190.t000257	92.68	5.03	−4.20	0	0
	BRI1	29,950.t000019	0.13	79.08	9.23	0	0
	BRI1	29,820.t000032	25.76	0.34	−6.23	0	0
	BKI1	30,170.t000310	5.19	0.55	−3.23	8.68E-19	1.98E-18
	BSK	30,190.t000453	42.70	12.11	−1.82	6.44E-97	3.60E-96
	BSU1	30,128.t000269	40.56	19.45	−1.06	1.46E-100	8.36E-100
	BZR1/2	29,646.t000010	120.71	43.54	−1.47	3.29E-232	3.79E-231
	TCH4	30,041.t000001	37.08	6.77	−2.45	2.06E-91	1.10E-90
	CYCD3	29,168.t000023	16.16	2.08	−2.96	4.11E-93	2.23E-92

RPKM, Reads Per kb per Million reads, represented the expression level of a gene

FC, the log2 fold changes of ZB306/ZB107

FDR, False Discovery Rate, a method to determine the appropriate threshold of the P-value in multiple test and analysis

### Correlations between proteome and transcriptome data

**Fig. 8:**
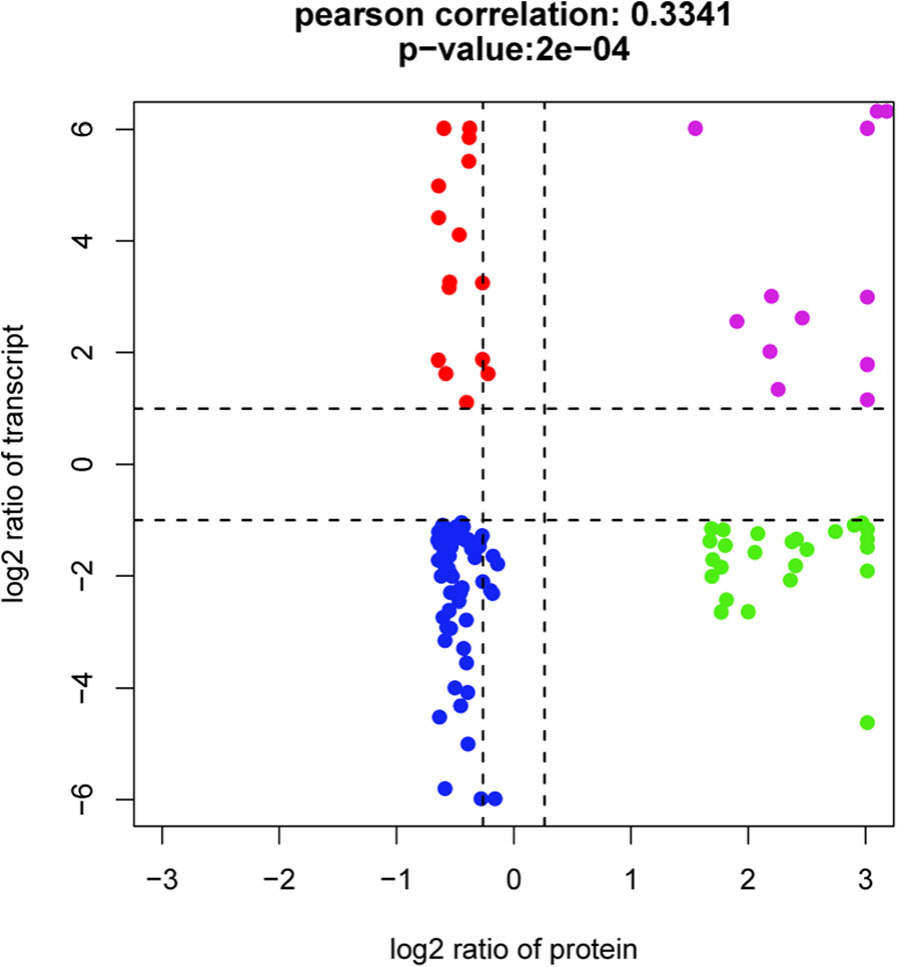
Relationship patterns of all DAPs and their corresponding genes. In the diagram, the *x*-axis is the protein species abundance and the *y*-axis is the gene expression. Each round dot denotes a log2 transcript ratio and a log2 protein ratio. Purple represents up-regulated genes and increased-abundance protein species; blue indicates down-regulated genes and decreased-abundance protein species; red indicates up-regulated genes and decreased-abundance protein species; and green indicates down-regulated genes and increased-abundance protein species

A global correlation analysis between DAPs and their corresponding transcripts was conducted, resulting in a low Pearson correlation coefficient (*r* = 0.334, *P* = 2e-04) (Fig. 8). The scatter plot analysis showed that 12 genes (indicated by the purple dot) were up-regulated at both the transcript and protein levels, while 68 genes (indicated by the blue dot) were down-regulated in small-seed ZB306 (Additional file 10: Table S6). These observations revealed that protein species abundances were determined by the corresponding mRNA expression levels, reflecting a strong correlation between transcripts and protein species. Among these down-regulated genes at both mRNA and protein levels, 15 genes were involved in carbohydrate metabolism (Additional file 11: Figure S5), such as CS (30,226.t000050), SUS (29,739.t000129), and pyruvate kinase (30,131.t000450); and 12 genes were related to protein metabolism, e.g., SRPL32 (29,070.t000002), SRPL8 (29,743.t000009), and SAMS (30,078.t000071). Our results clearly indicated that these genes involved in carbohydrate and protein metabolism were coordinately regulated at both transcript and protein levels and that seed-filling affects seed size/weight. Furthermore, 40 genes showed opposite trends at the proteomic and transcriptomic levels (Additional file 10: Table S6), implying that post-transcriptional and/or post-translational modifications might play an important role in determining protein species abundances. A total of 15 genes were up-regulated in mRNA levels and decreased in protein species abundances (indicated by the red dot), while 25 genes were significantly down-regulated in mRNA levels and increased in protein species abundances (indicated by the green dot in Fig. 8), e.g., SRPL12 (29,588.t000012), SRPL19 (30,167.t000025), and SRPL23 (29,805.t000016). It is known that ribosomal proteins play critical roles in numerous essential cell activities in plants, such as controlling many developmental programs through translational regulation or post-translational modifications in *Arabidopsis* [30]. Thus, changes in gene expression might not always adequately reflect proteins levels, and the post-transcriptional or post-translational modifications should not be ignored. Taken together, our data indicate that the major differences between large-seed ZB107 and small-seed ZB306 were found in carbohydrate and protein metabolism at both protein and transcript levels, while the differences in post-transcriptional or post-translational modifications may also exert influence on the seed-filling process.

### Validation of selected DAPs/DEGs by qRT-PCR analyses

**Fig. 9:**
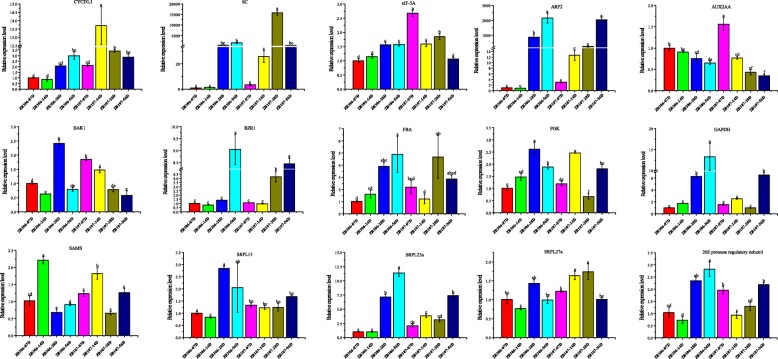
Expression patterns of genes related to the formation of seed size in ZB107 and ZB306 during seed development based on qRT-PCR. CYCD1;1, D1-type cyclin (29,168.t000023); SC, serine carboxypeptidase (29,745.t000011); eIF-5A, eukaryotic translation initiation factor 5a (29,687.t000001); ARF2 (29,647.t000040); AUX|IAA, auxin/indole-3-acetic acid (28,179.t000006); BAK1, BRI1-associated receptor kinase 1 (30,190.t000257); BZR1, BR-activated transcription factor BRASSINAZOLE-RESISTANT1 (29,646.t000010); FBA, fructose-bisphosphate aldolase (29,660.t000032); PGK, phosphoglycerate kinase (30,169.t000073); GAPDH, glyceraldehyde 3-phosphate dehydrogenase (30,169.t000047); SAM, S-adenosylmethionine synthetase (30,128.t000431); SRPL13, 60S ribosomal protein L13 (30,071.t000004); SRPL23a, 60S ribosomal protein L23a (28,180.t000008); RPL27a, 60S ribosomal protein L27a (30,147.t000153); and 26S protease regulatory subunit, 29,739.t000101. Expression levels were calculated by the 2^−∆∆CT^ method against the control gene expression. Three biological replicates for each gene were included, and the values of gene expression were shown as mean ± SD. Different lowercase letters indicate significant differences. Data were analyzed with one-way ANOVA and Tukey’s multiple comparison test, *p* < 0.05

To confirm the reliability of comparative proteomic and transcriptomic analysis, expression patterns of the 15 candidate DAPs/DEGs were detected at different developmental stages in ZB107 and ZB306 based on qRT-PCR (Fig. 9). For cell division, the expression level of CYCD1;1 was significantly higher in large-seed ZB107 than in small-seed ZB306, especially at 14 DAF, which suggested that CYCD1;1 regulates cell number mainly in the early stage of seed development. Similarly, the relative gene expression of eIF-5A was significantly higher in ZB107 than ZB306 from 7 to 28 DAF, consistent with the pattern of transcriptomic and proteomic analysis. Moreover, the gene expression of ARF2 and BZR1 increased gradually, whereas the expressions of AUX|IAA and BAK1 were down-regulated in both ZB107 and ZB306 from 7 to 56 DAF. In particular, at the early stage, the expressions of AUX|IAA and BAK1 were significantly higher in ZB107 compared to ZB306 (at 7 DAF), but at the middle stage (28 DAF), the expressions of ARF2, AUX|IAA and BAK1 were significantly lower in ZB107 than that in ZB306. For carbohydrate metabolism, the expression levels of PGK (phosphoglycerate kinase) at 14 DAF were higher in ZB107 than that in ZB306, indicating that differences in seed-filling mainly occurred in the early developmental stage. For protein metabolism and synthesis, the expression levels of the SRPL23a, SRPL27a, and 26S protease regulatory subunits were higher in ZB107 than that in ZB306 at 14 DAF, while genes encoding SAMS, SRPL13, SRPL23a, and 26S protease regulatory subunits were down-regulated in ZB107 at 28 DAF, to the extent that the expressions of these genes were not always correlated with their protein species abundances. All these results not only confirm the accuracy and reliability of the DAPs/DEGs involved in seed size formation, but also further illustrate that cell division, carbohydrate metabolism, and protein synthesis enact important roles in the formation of seed size.

## Discussion

### Comparative analysis of proteomic techniques for protein species identification

In recent years, many proteomic studies have been conducted on the metabolic processes in castor bean seeds, e.g., we have learned more about the processes that manage carbon assimilation during the seed-filling phase of seed development, gained new insights into the biosynthesis of fatty acids, and discovered further mechanisms that are responsible for the biosynthesis of storage proteins during seed development and germination [6, 18, 22, 31]. However, our current study focuses on addressing potential mechanisms behind the formation of seed size through the comparison of protein and transcript profiles of large-seed ZB107 and small-seed ZB306 during seed development. A total of 1416 protein species were identified from developing castor seeds using the iTRAQ method combined with LC-MS/MS analysis, 866 of which were newly identified in this study. It was well known that different proteomic approaches applied for protein species identification often leaded to variable results [32]. The iTRAQ labeling method used in the present study is more sensitive and powerful than the protein fractionation of 2-DGE or SDS-PAGE methods for protein species identification. Additionally, the iTRAQ method has been widely applied to investigate the potential molecular mechanisms that underlie seed development and the accumulation of storage reserves in developing seeds, such as in *Arabidopsis* [33], *Citrus sinensis* [34], and rice [35]. However, the largest number of protein species was observed in this study compared to previous studies, because the whole seeds from three different stages were used in this study. In short, our proteomic analysis will provide important information on seed development of castor bean.

### Proteomic identification of cell division and seed-filling related protein species

Castor bean typically has endospermic seeds, whose size is largely determined by the endosperm volume. In this study, we discovered that the endosperm size of castor bean was dependent on cell number rather than cell size, which was similar to the determination of grain size in rice and maize [36, 37]. Our proteomic analyses reveal that cell division and diverse metabolic processes play crucial roles in controlling the different seed size of ZB107 and ZB306. In particular, we found that ARP (30,190.t000561) exhibited different protein species abundances between large-seed ZB107 and small-seed ZB306, and it was reported that ARP in Chinese cabbage regulated the cell division of different tissues [38]. In addition, SC (29,745.t000011) and eIF-5A (29,687.t000001) exhibited higher protein species and transcript abundances in large-seed ZB107 than small-seed ZB306, and it had been demonstrated that both SC and eIF-5A played critical roles in regulating cell division and controlling cell cycles in rice [39]. Consequently, these protein species might be the putative controllers that regulate cell division and determine the formation of seed size/weight in castor bean.

It is known that primary metabolism mainly occurs during the early stage of seed development, and many protein species involved in carbohydrate and protein metabolism have been identified in this study. Central carbon metabolism (including glycolysis and TCA cycle) is a main physiological process during seed-filling [40]. A total of 18 protein species involved in carbohydrate metabolism showed higher protein species abundances in large-seed ZB107 than in small-seed ZB306. For instance, You et al. found that the protein species abundances of FBA (29,660.t000032), PGK (30,169.t000073), and glucose-6-phosphate isomerase (GPI, 30170.t000437) involved in glycolysis were higher in larger grains than smaller grains during the grain-filling period of rice [13]. Thus, the above protein species, with differential abundances in carbohydrate metabolism during seed development, might be involved in regulating the formation of seed size by affecting seed-filling in castor bean. Additionally, the seeds of castor bean contain nearly 46–55% oil by weight, and nearly 90% of the oil is hydroxy fatty acid, while several enzymes in the TCA cycle, such as aconitase, malic enzyme, and CS also participated in fatty acid metabolism through the glyoxylate cycle [41]. We identified that the GAPDH protein (30,169.t000047), a key enzyme for activating fatty acid biosynthesis in glycolysis [42], had higher protein species abundances in large-seed ZB107 compared to small-seed ZB306. A previous study indicated that the activity and stability of GAPDH were critical in controlling lipid biosynthesis during the seed development of castor bean [43]. In addition, protein metabolism plays a critical role in regulating embryogenesis/endosperm genesis and storage protein biosynthesis [44]. We identified that the protein species abundances of SRPL13 (30,071.t000004), SRPL20 (30,147.t000462), SRPL8 (29,743.t000009), SRPL23a (28,180.t000008), and SRPL27a (30,147.t000153) were significantly increased in large-seed ZB107. Particularly, SRPL27a is critical for controlling seed development by regulating cell division and cell cycle progression in *Arabidopsis* [45, 46], another ribosomal L18 protein, HEART STOPPER (HES), has been shown to be essential during the early seed development of *Arabidopsis* via influencing cell division and cell differentiation [47]. The protein species abundance of the 26S protease regulatory subunit (29,739.t000101) was significantly increased in ZB107, which played a pivotal role in regulating cell cycle and differentiation during embryogenesis in *Arabidopsis* [48]. Moreover, xylem serine proteinase (29,986.t000074), an enzyme critical in the regulation of xylem biosynthesis [49], was increased in large-seed ZB107. Likewise, xylem serine proteinase was also involved in the formation of seed coat in *Arabidopsis* and *Glycine max* [50]. Taken together, these DAPs involved in the metabolism or biosynthesis of carbohydrates, proteins, lipids, and xylem might be putative controllers directly or indirectly influencing the formation of seed size/weight in castor bean. Further research should investigate the molecular mechanisms of these proteins underlying seed size variation.

### Transcriptomic identification of key genes involved in seed size formation

Comparative transcriptome analysis revealed that a total of 120 DEGs were involved in cell development, cell division, cell growth, and cell cycle control, suggesting that these genes might participate in controlling seed size through regulating cell division or cell cycle. In addition, a kinesin gene (29,171.t000006) involved in cell division was up-regulated in large-seed ZB107, which could potentially determine seed size by the regulation of cell number in *Nicotiana tabacum* [51]. Similarly, TT1 (30,169.t000194) in cell development was also up-regulated in large-seed ZB107, and it has been demonstrated that BnTT1 not only regulated flavonoid biosynthesis, but also affected seed size and fatty acid composition in *Brassica napus* [52, 53]. In carbohydrate metabolism, the gene expression levels of SUS (29,739.t000129, 29,951.t000003, and 29,660.t000014) were higher, suggesting that the content of hexoses is higher in large-seed ZB107 than in small-seed ZB306. The high levels of hexoses could maintain cell division and cell expansion during the embryogenesis of *Vicia faba*, and overexpression of a potato SUS gene in maize seeds could increase the starch content [54, 55]. Furthermore, it has been proved that the activity of SUS is positively correlated with seed size in chickpea [56]. Additionally, several plant hormones such as auxin, cytokinin, and BR could potentially regulate seed size through cell development (Fig. 7). For example, the expression of ARF2 (29,647.t000040) was lower in large-seed ZB107, and it could negatively regulate seed size via repression of cell division in the integument region of *Arabidopsis* seed [57]. It is likely that ARF2 may be responsible for determining seed size in castor bean via controlling the extension of the seed coat. Similarly, cytokinins have also been reported to affect seed size through maternal or zygotic tissues [58]. AHP4 (29,912.t000167) was down-regulated in large-seed ZB107, which was a negative regulator of cytokinin signaling [59]. Moreover, AHP4 has been reported to affect seed size, vascular development, and other aspects of plant development in wheat [60]. The BR-activated transcription factor BRASSINAZOLE-RESISTANT1 (BZR1, 29,646.t000010) could potentially regulate seed size and shape via influencing specific processes in the integument, endosperm, and embryo development in *Arabidopsis* [61]. The expression patterns of these genes involved in cell division, signal transduction of plant hormones, carbohydrate metabolism, and protein metabolism were confirmed by qRT-PCR during the seed development of ZB107 and ZB306. Hence, we suggest that the above-mentioned processes are responsible for the seed size/weight variation in castor bean.

### The global relationship between developing seeds proteome and transcriptome

Recent advances in proteome and transcriptome profiling technologies have provided new opportunities to integrate protein abundance with gene expression in the analysis of the molecular mechanisms that underlie the formation of seed size in castor bean at multiple levels. The consistency between protein species abundances and mRNA expressions in carbohydrate and protein metabolism indicates that gene expression determines the corresponding protein species abundances. In the process of protein metabolism, highly expressed genes, SAMS (30,078.t000071/30128.t000431), were always rich in protein species abundances and their transcript levels and protein species abundances were higher in large-seed ZB107 compared to small-seed ZB306 (Fig. 4d). A previous study showed that overexpression of SAMS in transgenic tobacco increased the number and weight of seeds [62]. Occasionally, there is a poor correlation between protein species abundances and mRNA levels, which has been demonstrated in previous studies of developing cotton fibers, and highlights the importance of post-transcriptional and/or post-translational regulation [63]. Post-transcriptional and post-translational modifications could impact transcript and protein stability [64] as well as influence the translation and metabolism processes. Ribosomal proteins played an essential role in translation, and several ribosomal proteins were the targets of post-translational modifications, which led to the inconsistency of mRNA levels and protein species abundances [65]. For example, the protein species abundances of SRPS12/19/23 were increased in small-seed ZB306 compared to large-seed ZB107, while their gene expressions exhibited the opposite pattern (Fig. 4c). So far, almost no research has focused on how post-transcriptional modifications and/or post-translational modifications affect seed size. In future studies, numerous post-transcriptional and post-translational regulatory processes should be considered when referring to the proteomics of the molecular basis behind seed development.

## Conclusions

Castor bean seeds are typically endospermic in dicotyledons because of their persistent endosperms in maturity. In this study, histological observation revealed that seed size was determined by cell number rather than cell size at the cellular level in the endosperm of castor bean. Combining with comparative transcriptomic and proteomic analyses of developing seeds from two inbred varieties of large-seed ZB107 and small-seed ZB306, we identified that most of DAPs/DEGs were functionally involved in cell division and storage reservoir accumulation, consistent with the formation of seed size in volume and weight. These identified DAPs/DEGs may play a critical role in governing the formation of seed size in castor bean. The correlative analysis between proteomic and transcriptomic data showed that many genes involved in cell division, carbohydrate metabolism, and protein metabolism were correlatively regulated at both transcript and protein levels, though the Pearson correlation coefficient was low (*r* = 0.334). These data could provide a crucial resource to further understand the molecular mechanisms governing seed size/weight in endospermic seeds of castor bean. We hope this research will help us to unravel the genetic foundation in the improvement of crop yields by increasing the seed size.

## Methods

### Plant materials and sampling

The seeds of castor bean elite inbred lines ZB107 (large-seed) and ZB306 (small-seed) were the primary materials in this study. ZB107 and ZB306 were both grown in the Xishuangbanna Tropical Botanical Garden of the Chinese Academy of Science (Menglun Town, Mengla County, Yunnan Province, China; 21°56′N, 101°15′E, 600 m elevation). This region is characterized by an average temperature of 21 °C~ 22 °C. The seeds of ZB107 were collected 7, 21, 35, 49, and 63 DAF, corresponding to ZB306 at 7, 14, 28, 42, and 56 DAF. Each time, at least twenty seeds from five independent plants were used for experiments, and the same tests were performed across three biological replicates. Samples for iTRAQ and RNA-seq analyses were immediately frozen in liquid nitrogen and stored at − 80 °C, while the remaining samples were used for measuring fresh and dry weight.

### Seed weight and protein content measurements

The individual seeds of all harvested materials were immediately weighed for average fresh weight using an analytical balance (TP-214; Denver Instrument, USA), while average dry weight of individual seeds was measured after drying samples at 65 °C for 48 h in an oven. Protein content was determined on the basis of dry mass using a Kjelmaster K-375 Automatic Steam Distillation System as previously described [66]. Three seeds from three independent plants were successively used to examine the FW, DW, and protein content as one biological replicate and average values were calculated from three biological replicates.

### Morphological and cellular analysis

To determine seed size, we photographed the projective area of mature dry seeds and endosperm. Ten seeds were freshly isolated from five independent plants of ZB107 at 63 DAF and ZB306 at 56 DAF. Seed coat and embryo were both dissected from seeds in order to measure the cell size of the endosperm. Endosperms were trimmed into appropriately sized samples, fixed in a formaldehyde-acetic acid solution for no less than 48 h at room temperature, dehydrated in a series of ethanol in ascending concentration, cleared in xylene, and embedded in paraffin as previously described by Nogueira et al. [18] with some modifications. Each endosperm was divided into internal, middle, and external parts to measure cell size, and each part was sectioned at 5~10 μm thickness under a CM3050S rotary microtome (Leica, Germany). These sections were stained with 0.05% toluidine blue to visualize the cell size on a Leica microscope (DM5500B, Bensheim, Germany). At least twenty images from three parts of the endosperm were photographed using Leica Microsystems (DFC450C). Endosperm area and endosperm cell area were analyzed using Image J software.

### Protein preparation and iTRAQ labeling

We used whole developing seeds of ZB107 at 21, 35, and 63 DAF as well as ZB306 at 14, 28, and 56 DAF for further proteomics analysis (Additional file 12: Figure S6). Proteins were extracted from each sample with three independent replicates. Protein concentration was determined by 2D Quant kit (GE Healthcare), and verified using SDS-PAGE. Equal amounts of three protein samples from ZB107 were mixed as ‘large-seed’, and equal amounts of three protein samples from ZB306 were mixed as ‘small-seed’. The protein samples (100 μg) of large-seed ZB107 samples and small-seed ZB306 samples were digested with trypsin, then the peptides were labeled with 117 and 121 tags, respectively, using 8-plex iTRAQ reagents. All samples were pooled with equal fractions and dried in a vacuum centrifuge.

### SCX fractionation

The iTRAQ-labeled peptides were fractionated using Agilent 1200 HPLC with strong cation exchange (SCX) chromatography. The dried iTRAQ-labeled peptides were diluted in buffer A (10 mM KH_2_PO_4_, 25% ACN, pH 3.0). The HPLC was performed at a flow rate of 1.0 mL/min with a 50 min HPLC gradient consisting of 100% buffer A for 5 min, 0~20% buffer B (10 mM KH_2_PO_4_, 25% ACN, 500 mM KCL, pH 3.0) for 15 min, 20%~ 40% buffer B for 10 min, 40%~ 100% buffer B for 10 min, and 100% buffer A for 10 min. The eluted peptides were collected and combined into twelve fractions. Each fraction was dried in a vacuum dryer and re-suspended in 0.1% formic acid (FA) for LC-MS/MS analysis.

### LC-MS/MS analysis

The LC fractions were analyzed using a Triple TOF 5600 mass spectrometer system equipped with nanoflow reversed-phase liquid chromatography (RPLC) system (AB SCIEX). The peptide fractions were loaded onto a nanobored C18 column at a flow rate of 0.20 μL/min. An elution gradient of 5%~ 40% ACN (0.1% FA) within a 120 min gradient was used. The mass spectroscopy data were acquired using an ion spray voltage of 2.5 kV, curtain gas of 30 PSI, nebulizer gas of 5 PSI, and an interface heater temperature of 150 °C. We used an information-dependent acquisition (IDA) mode to acquire MS/MS data. Survey scans were acquired in 250 ms intervals, and as many as 35 product ion scans were collected with a 20 s exclusion window and a total cycle time of 2.5 s. A rolling collision energy setting was applied to all precursor ions for CID. The data acquisition rate was 4 s per spectrum.

### Proteomic data analysis

The raw data were converted into MGF files using Proteome Discoverer software (Thermo Scientific, USA). The identification of protein species was performed using the Mascot software (Matrix Science) against the *Ricinus communis* database (ftp://ftp.tigr.org/pub/data/castorbean/release_0.1/). The following criteria were applied: iTRAQ 8-plex was chosen for quantification with unique peptides, peptide mass tolerance value was set at 20 ppm, and the tolerance of MS/MS was set at 0.6 Da. The quantitative protein species ratios were calculated and normalized by the median ratio of only unique peptides. For the quantification of protein species, a mass-to-charge ratio (m/z) of 117 was performed as the control sample in accordance with the peak area of m/z 121 reporter ions. A sequest score of HT > 0 for authentic proteins and ≥ 1 for unique peptides were used as the screening criteria. The miss values were maintained with the null values. Protein species with a fold change ≥1.5 or ≤ 0.67 and a *p*-value <0.05 in all three replicates were determined as differentially abundant protein species (DAPs).

The functions of all the protein species were annotated using the NCBI nr and SwissProt/Uniprot databases and further analyzed using the Cluster of Orthologous Groups of proteins (COG), Gene Ontology (GO), and the Kyoto Encyclopedia of Genes and Genomes (KEGG) databases. These identified DAPs were subjected to GO and KEGG enrichment analyses. Hierarchical clustering of protein species between the two samples was visualized using MeV 4.9.0 software.

### Transcriptome sequencing and data analysis

Total RNA was extracted from the same samples used for the proteomics analysis with the RNAprep Pure Plant Kit (Tiangen, Beijing, China). RNA concentration and integrity were measured using the Agilent 2100 Bioanalyzer (Agilent Technology, USA). Two RNA-seq libraries (ZB107 and ZB306) were constructed and sequenced on an Illumina HiSeq 2000 platform. After removing low-quality reads, the sequencing data were mapped to the castor bean reference genome using SOAP2 with default parameters [67]. Reads per kilobase of exon model per million mapped reads (RPKM) were used to estimate the expression levels of genes. Differentially expressed genes (DEGs) between ZB306 and ZB107 were identified with a FDR ≤ 0.01 and |log2 (fold change) | ≥ 1. GO and KEGG Pathway enrichment analysis for DEGs were carried out by Omicshare Tools (http://www.omicshare.com/tools/Home/Soft/getsoft). To identify the *cis*-elements in the promoter of genes involved in the hormone signal transduction pathways, 1500 bp upstream sequences of genes were extracted from reference genome and *cis*-elements were detected by querying the PlantCARE database (http://bioinformatics.psb.ugent.be/webtools/plantcare/html/).

### Correlation analyses of transcriptome and proteome

To investigate the concordance between transcriptome and proteome during the seed development of ZB107 and ZB306, a correlation analysis was performed based on DAPs and their corresponding transcripts. The Pearson’s correlation coefficient was calculated for these data and scatter plots were created with the log2-transformed ratios of transcripts and corresponding protein species abundances.

### qRT-PCR validation

We selected fifteen genes to validate their expression patterns in the ZB306 and ZB107 seeds when at different developmental stages using qRT-PCR. Total RNAs were extracted independently from eight samples, including the seeds of ZB306 at 7, 14, 28, and 56 DAF along with the corresponding seeds of ZB107 at 7, 21, 35, and 63 DAF. At this stage, to facilitate the description of the seed development periods, we used the period of ZB306 to represent the corresponding developmental period of ZB107. The cDNA was synthesized with 1 μg total RNA using the TransScript All-in-One First-Strand cDNA Synthesis SuperMix for qPCR kit (TransGen Biotech, Beijing, China), and qRT-PCR was carried out according to our previous studies [21]. We performed testing across three biological replicates for each gene and each experiment. The relative expression values for each gene in each RNA sample were mean ± SD. The primers used for qRT-PCR were designed using Primer 3 web and were presented in Additional file 13: Table S7.

### Statistical analysis

For all statistical significance analyses, at least three biological replicates were used for ZB107 and ZB306. Student’s *t*-test and one-way analysis of variance (ANOVA) followed by Tukey’s multiple comparison test (*P* < 0.05) was performed using SPSS18.0. The following asterisks indicate the results of significance testing: *p < 0.05 and ** *p* < 0.01. Different lowercase letters in the graphs indicate significant differences. Data represent mean values and error bars are SD.

## Supplementary information

**Additional file 1: Table S1.** Numbers of spectra, peptides, and protein species identified in the iTRAQ analysis

**Additional file 2: Table S2.** Peptide sequences of the identified protein species (XLS 904 kb)

**Additional file 3: Figure S1.** The COG functional category analysis of all proteins.

**Additional file 4: Table S3.** Eight protein species involved in cell cycle control, cell division, chromosome partitioning

**Additional file 5: Figure S2.** GO categories for all protein species identify in both ZB107 and ZB306.

**Additional file 6: Table S4.** Total list of the differentially abundant protein species (XLS 128 kb)

**Additional file 7: Figure S3.** Schematic representation of the DAPs and DEGs involved in carbohydrate metabolism of castor bean seed.

**Additional file 8: Table S5.** Gene functional classes of protein species only detected in our comparative proteomics

**Additional file 9: Figure S4.** *Cis*-element analysis of promoter sequences of genes in hormone signal transduction pathways.

**Additional file 10: Table S6.** Correlation analysis of the DAPs and the corresponding genes

**Additional file 11: Figure S5.** The KEGG annotation of genes that were correlated between protein and transcript levels.

**Additional file 12: Figure S6.** Workflow of identify DAPs/DEGs between large-seed ZB107 and small-seed ZB306.

**Additional file 13: Table S7.** Summary of primers used in this study.

## Data Availability

The data sets are included within the article and its Additional files. The sequencing data used for this study have been deposited into the NCBI Sequence Read Archive (SRA, http://www.ncbi.nlm.nih.gov/Traces/sra) database under the accession number of SRR1313230 and SRR1313233.

## Abbreviations

AHP4: Arabidopsis histidine-containing phosphotransfer protein 4
ARF2: Auxin response factor 2
ARP: Auxin-repressed 12.5 kDa protein
AUX|IAA: Auxin/indole-3-acetic acid
BAK1: BRI1-associated receptor kinase 1
BRs: Brassinosteroids
BSK: BR-signaling kinase
BZR1: BRASSINAZOLE-RESISTANT1
CIGR: Chitin-inducible gibberellin-responsive protein
COG: Cluster of Orthologous Groups of proteins
CS: Citrate synthase
DAF: Days after fertilization
DAPs: Differentially abundant protein species
DEGs: Differentially expressed genes
DW: Dry weight
eIF-5A: Eukaryotic translation initiation factor 5a
FA: Formic acid
FBA: Fructose-bisphosphate aldolase
FW: Fresh weight
GAPDH: Glyceraldehyde 3-phosphate dehydrogenase
GO: Gene Ontology
GPI: Glucose-6-phosphate isomerase
IDA: Information-dependent acquisition
iTRAQ: Isobaric tags for relative and absolute quantification
KEGG: Kyoto Encyclopedia of Genes and Genomes
PGK: Phosphoglycerate kinase
PGM: Phosphoglucomutase
RPKM: Reads per kilobase of exon model per million mapped reads
RPLC: Reversed-phase liquid chromatography
SAMS: S-adenosylmethionine synthetase
SC: Serine carboxypeptidase
SUS: Sucrose synthase
TCA: Ttricarboxylic acid
TIR1: Transport inhibitor response 1 protein
TPI: Triosephosphate isomerase
TT1: TRANSPARENT TESTA 1 protein
