# Supplementary material for: Comparative proteomic and transcriptomic analyses provide new insight into the formation of seed size in castor bean
Source: BMC Plant Biol. 2020 Jan 30;20:48. doi: 10.1186/s12870-020-2249-1 (PMC6993385; doi:10.1186/s12870-020-2249-1)
Supplement: Supplementary file 3 — Additional file 3: Figure S1. The COG functional category analysis of all proteins. [file 12870_2020_2249_MOESM3_ESM.pdf]

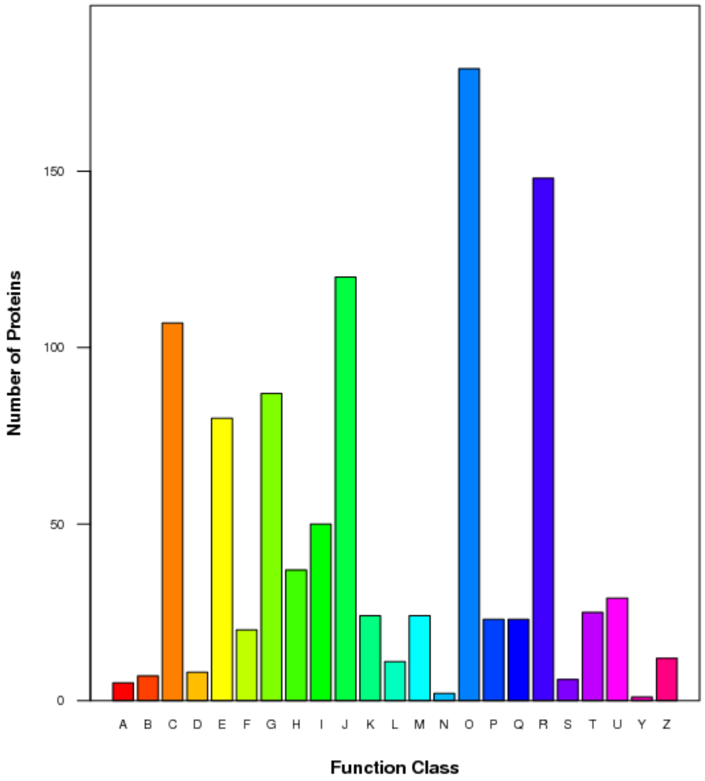

Fig S1. The COG functional category analysis of all proteins. Function classes. A: RNA processing and modification (number of proteins is 5, the percentage is 0.49%); B: Chromatin structure and dynamics (7, 0.68%); C: Energy production and conversion (107, 10.41%); D: Cell cycle control, cell division, chromosome partitioning (8, 0.78%); E: Amino acid transport and metabolism (80, 7.78%); F: Nucleotide transport and metabolism (20, 1.95%); G: Carbohydrate transport and metabolism (87, 8.46%); H: Coenzyme transport and metabolism (37, 3.60%); I: Lipid transport and metabolism (50, 4.86%); J: Translation, ribosomal structure and biogenesis (120, 11.67%); K: Transcription (24, 2.33%); L: Replication, recombination and repair (11, 1.07%); M: Cell wall/membrane/envelope biogenesis (24, 2.33%); N: Cell motility (2, 0.19%); O: Posttranslational modification, protein turnover, chaperones (179, 17.41%); P: Inorganic ion transport and metabolism (23, 2.24%); Q: Secondary metabolites biosynthesis, transport and catabolism (23, 2.24%); R: General function prediction only (148, 14.40%); S: Function unknown (6, 0.58%); T: Signal transduction mechanisms (25, 2.43%); U: Intracellular trafficking, secretion, and vesicular transport (29, 2.82%); Y: Nuclear structure (1, 0.10%); Z: Cytoskeleton (12, 1.17%)
