# Supplementary material for: Comparative proteomic and transcriptomic analyses provide new insight into the formation of seed size in castor bean
Source: BMC Plant Biol. 2020 Jan 30;20:48. doi: 10.1186/s12870-020-2249-1 (PMC6993385; doi:10.1186/s12870-020-2249-1)
Supplement: Supplementary file 5 — Additional file 5: Figure S2. GO categories for all protein species identify in both ZB107 and ZB306. [file 12870_2020_2249_MOESM5_ESM.pdf]

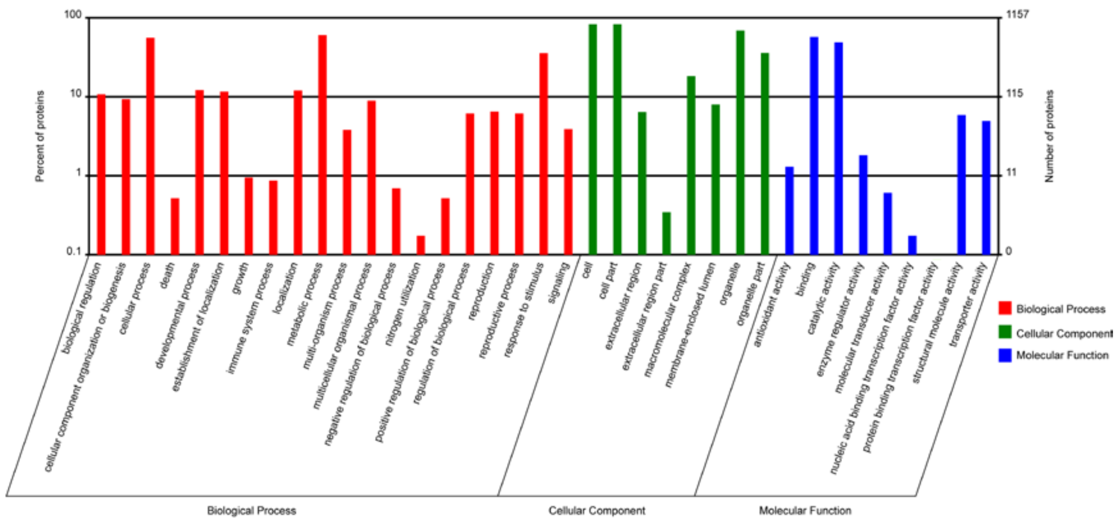

Fig S2. GO categories for all protein species identify in both ZB107 and ZB306. The x-axis represents each GO term. The left y-axis represents the percentage of genes for each function belonging to a main category of genes in that main category; the right y-axis represents the correspondent number of protein species.
