# Supplementary material for: Comparative proteomic and transcriptomic analyses provide new insight into the formation of seed size in castor bean
Source: BMC Plant Biol. 2020 Jan 30;20:48. doi: 10.1186/s12870-020-2249-1 (PMC6993385; doi:10.1186/s12870-020-2249-1)
Supplement: Supplementary file 7 — Additional file 7: Figure S3. Schematic representation of the DAPs and DEGs involved in carbohydrate metabolism of castor bean seed. [file 12870_2020_2249_MOESM7_ESM.pdf]

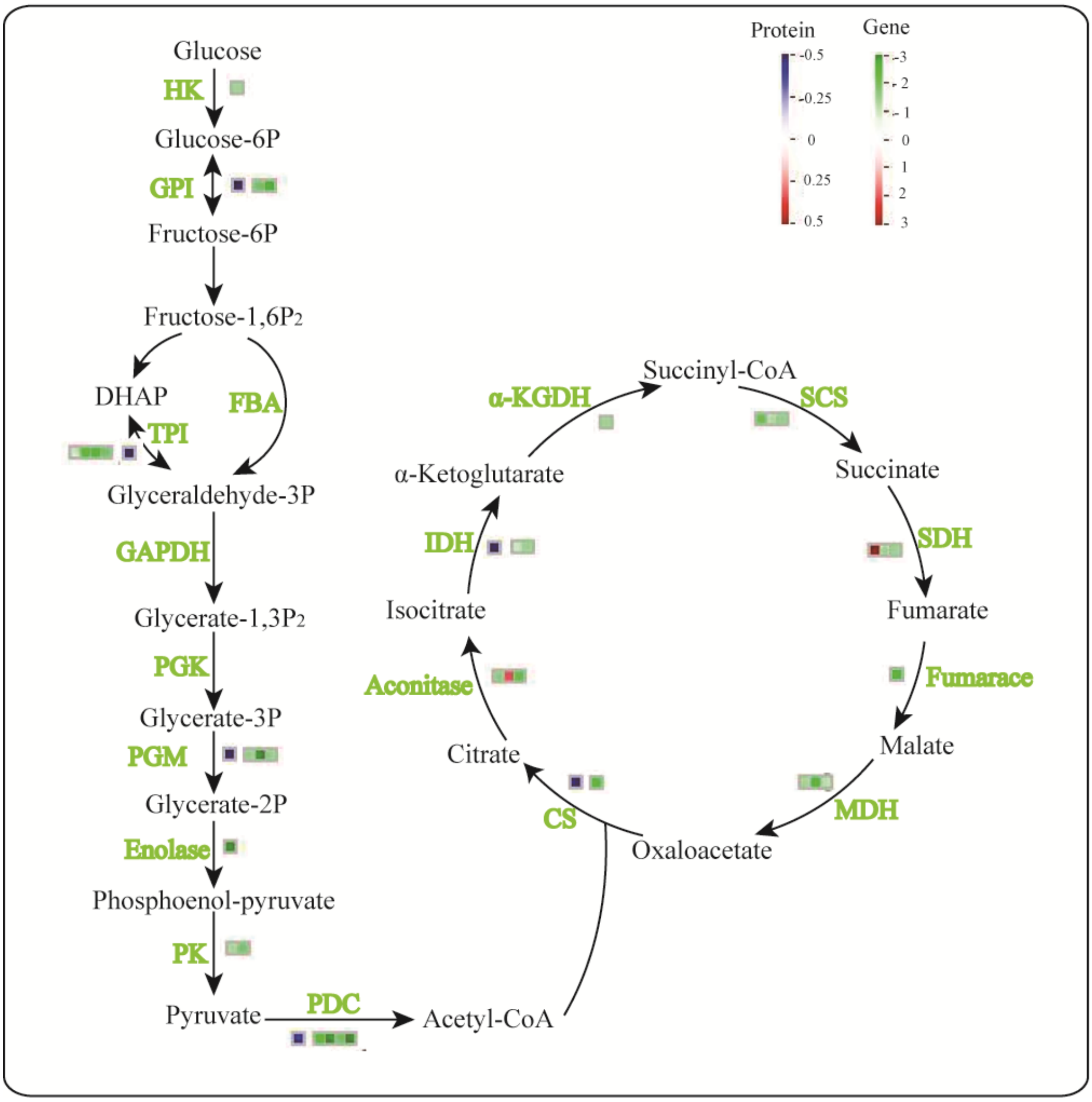

Fig S3. Schematic representation of the DAPs and DEGs involved in carbohydrate metabolism of castor bean seed. *Red* and *blue* indicate up- and down-regulated proteins, *red* and *green* indicate up- and down-regulated genes. HK, hexose kinase; GPI, glucose-6-phosphate isomerase; TPI, triosephosphate isomerase; PGM, phosphoglycerate mutase; PK, pyruvate kinase; PDC, pyruvate dehydrogenase; CS, citrate synthase; IDH, isocitrate dehydrogenase; α-KGDH, α-ketoglutarate dehydrogenase; SCS, succinyl CoA synthetase; SDH, succinate dehydrogenase; MDH, malate dehydrogenase.
