# Supplementary material for: Comparative proteomic and transcriptomic analyses provide new insight into the formation of seed size in castor bean
Source: BMC Plant Biol. 2020 Jan 30;20:48. doi: 10.1186/s12870-020-2249-1 (PMC6993385; doi:10.1186/s12870-020-2249-1)
Supplement: Supplementary file 9 — Additional file 9: Figure S4. Cis-element analysis of promoter sequences of genes in hormone signal transduction pathways. [file 12870_2020_2249_MOESM9_ESM.pdf]

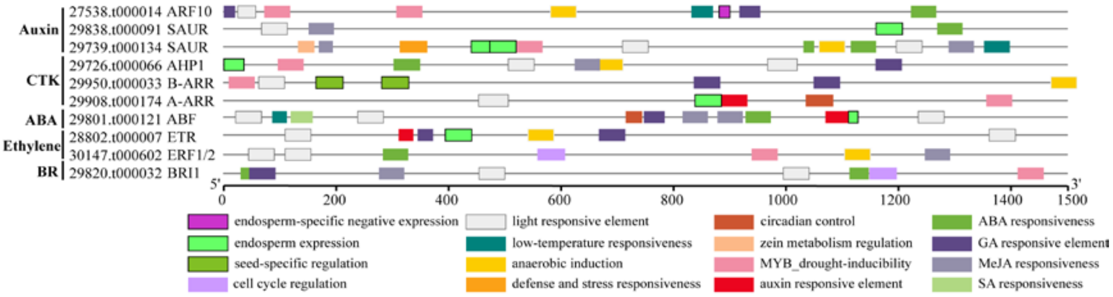

Fig S4. *Cis*-element analysis of promoter sequences of genes in hormone signal transduction pathways. Colored boxed represent different *cis*-element.
