# Supplementary figures and images for: Comparative proteomic and transcriptomic analyses provide new insight into the formation of seed size in castor bean
Source: BMC Plant Biol. 2020 Jan 30;20:48. doi: 10.1186/s12870-020-2249-1 (PMC6993385; doi:10.1186/s12870-020-2249-1)

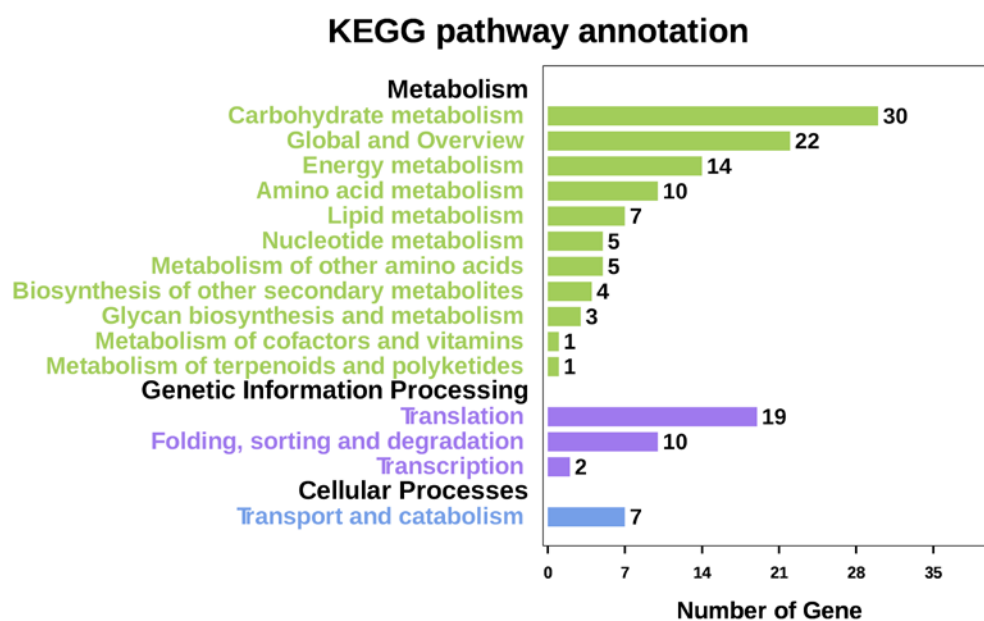

Fig S5. The KEGG annotation of genes that were correlated between protein and transcript levels.

Supplement: Supplementary file 11 — Additional file 11: Figure S5. The KEGG annotation of genes that were correlated between protein and transcript levels. [file 12870_2020_2249_MOESM11_ESM.pdf]

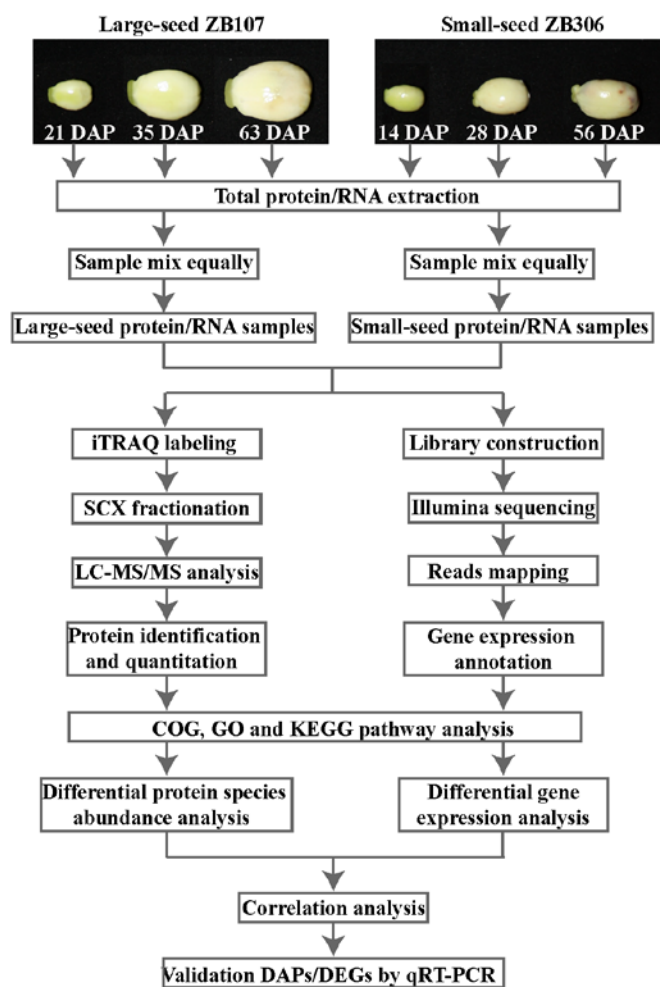

Fig S6. Workflow of identify DAPs/DEGs between large-seed ZB107 and small-seed ZB306.

Supplement: Supplementary file 12 — Additional file 12: Figure S6. Workflow of identify DAPs/DEGs between large-seed ZB107 and small-seed ZB306. [file 12870_2020_2249_MOESM12_ESM.pdf]
